# Development of a Health Impact Assessment Implementation Model: Enhancing Intersectoral Approaches in Tackling Health Inequalities- A Mixed Methods Study Protocol

**DOI:** 10.12688/hrbopenres.13873.2

**Published:** 2024-12-24

**Authors:** Monica O'Mullane, Tara Kenny, Kirsty Nash, Sheena M. McHugh, Paul Kavanagh, Katherine Smith

**Affiliations:** 1 School of Public Health, University College Cork, Cork, County Cork, Ireland; 2 Health Services Executive, Tullamore, County Offaly, Ireland; 3 School of Social Work and Social Policy, University of Strathclyde, Glasgow, Scotland, UK

**Keywords:** Health Impact Assessment, action research, implementation science, city planning, climate action

## Abstract

Public health research presents compelling evidence that health is socially determined. To address structural inequalities and inequities in health, public policies require intersectoral development and implementation. Health Impact Assessment (HIA) is an established approach for analysing potentially detrimental health impacts of policies, programmes, and projects, as well as potentially positive impacts and opportunities. National public health policy, *Healthy Ireland* (2013–2025), endorses an intersectoral whole-of-system approach to ensure that health is a central part of all relevant policy areas. HIA is endorsed in this policy as one way to drive this agenda. Synergising with this policy commitment for HIA, the all-island Institute of Public Health Ireland produced revised HIA guidance in 2021. Two HIAs will be carried out as part of this project, including one at a local policy level, addressing the Cork City Development Plan (2022–2028), and the second HIA at a national policy level, addressing the Irish Government’s Climate Action Plan (2024). The updated HIA guidance will be used in the conduct of these HIAs. This research project involves a co-creation of a Health Impact Assessment Implementation Model by employing an action research approach with implementation science frameworks to the conduct of the two HIAs. Therefore, the process of doing the HIAs will form the basis for the research study. In order to enhance meaningful community involvement in HIA in Ireland, the project will co-create a Community Engagement Toolkit for HIA. This Model will strengthen researcher, policy actor, practitioner, community, and voluntary sector capacity to collaboratively develop and implement intersectoral and equitable policy responses to major population health issues.

## Introduction

Public health research has shown that our health and wellbeing are affected by the circumstances into which we are born, grow, live, work and age. These wider social, economic, political and environmental circumstances are a greater determinant of health status than individual factors and behaviours ( CSDH, 2008). Health inequalities, which are a result of systemic inequities across these wider determinants of health on population groups, require intersectoral action from beyond health sector contexts ( Marmot, 2017; Solar *et al.*, 2023). In an Irish context, published by the government Department (Ministry) of Health, the *Healthy Ireland* national survey (2019) reaffirmed that people living in deprived areas are more likely to suffer from health conditions compared to those in wealthier areas ( DOH, 2019). Those who are employed and higher educated are more likely to report 'good or very good' health ( DOH, 2023; Duffy *et al.*, 2022).

The impact of the COVID-19 pandemic in Ireland was experienced more negatively by marginalised communities in society ( EAPN, 2020). Clearly, what is needed is a tangible way for all policy sectors to work collaboratively in improving population health and tackling health inequalities.

HIA is a process that encompasses a set of tools to identify best possible outcomes related to categories of objectives needed to strengthen the promotion of health and health equity. HIA is “a process which systematically judges the potential, and sometimes unintended, effects of a project, programme, plan, policy, or strategy on the health of a population and the distribution of those effects within the population” ( Winkler *et al.*, 2021: 3). The WHO Gothenberg Consensus paper (1999) identified four core values of HIA, which continue to underpin the process to this day, namely, democracy, equity, ethical use of evidence and sustainable development. HIA is conceptually underpinned by the wider determinants of health ( Solar & Irwin, 2010). It is through the examination of this aforementioned ‘distribution of effects’ across identified population groups, and collection of data within HIA, that health equity can be improved, and health inequalities tackled. HIA is an established approach for implementing Health in All Policies ( Bekker, 2007; DoH, 2013; Green *et al.*, 2021b; Pyper *et al.*, 2021) in order to tackle health inequalities ( Douglas & Scott-Samuel, 2001). It can strengthen the Health in All Policies approach to strengthening the co-benefits across health, economic and environmental improvements ( Greer *et al.*, 2024). HIA, as well as providing formal evidence of potential health-related outcomes across sectors, has been shown to broaden the set of issues under scrutiny, the types of decisions assessed, and the range of actors involved ( Green *et al.*, 2021a). Lynch *et al.* (2023) advocate for governmental support of HIA as a way to implement Health in All Policies and improve health equity across policy domains.

Despite continued HIA practice and policy for more than two decades, ( O'Mullane, 2013; Rogerson *et al.*, 2020), HIA implementation- the doing or practice of HIA- remains ad hoc in many jurisdictions. HIA practice and policy in Ireland has been stopping and starting since its initial and strongest policy endorsement to date, in the public health policy *Quality and Fairness: A Health System for you*, published by the then Department (government Ministry) of Health and Children (DOH&C) ( 2001). HIA has been criticised over the years, predominantly for adding to the work burden of statutory stakeholders ( Linzalone *et al.*, 2018), becoming a tick-the-box exercise, and not aligning the informational pathway from the HIA with the relevant respective policies, projects or programmes. In Ireland, research found it often did not have impact beyond the health sector because of poor intersectoral collaborations ( O'Mullane, 2015). This finding concurs with a review of HIA progress in Ireland, which concluded that an implementation gap exists in relation to surmounting cultural and professional boundaries and acceptance of a joint intersectoral approach ( Gillespie & McIldoon, 2009). Other challenges for implementing HIA identified in the review include time, capacity, and resource limitations; issues regarding roles and responsibilities; and the impression of policy stakeholders is that HIA is a complicated process. These challenges are deemed surmountable ( Gillespie & McIldoon, 2009) by way of adopting a triadic approach to enhancing HIA implementation in Ireland by a) improving the HIA implementation process; b) providing HIA capacity-building; c) garnering political will and leadership. HIA-IM will develop a HIA implementation model addressing points a and c; it will also indirectly inform the Institute of Public Health HIA programme of capacity-building (b) ( IPH, 2020).

The premise of the project is to explore the doing or implementation of two HIAs with a view to developing a HIA implementation model that includes strategies to overcome barriers in the doing of HIA as identified in the two HIAs. We chose to use two implementation science (IS) frameworks (NPT and CFIR) in developing this HIA implementation model because of the value those IS frameworks bring to this field of research. NPT focuses more on the process of implementing whereas CFIR focuses more on the determinant hindering and enabling factors affecting the implementation or doing of HIA. Both frameworks complement one another in creating data to build the HIA implementation model, given their differing emphasis on process (NPT) and factors affecting or determining the implementation or doing of HIA (CFIR).

The rationale for choosing the action research design approach, is to ensure the creation of the implementation model is underpinned by an iterative development of the implementation model, involves learning, reflection and action in the HIA approach of those involved in the HIA Steering Groups.

This multifocal approach to enhancing Health in All Policies through HIA implementation has been demonstrated in other countries, including Wales ( Green *et al.*, 2020), Scotland ( Douglas *et al.*, 2020), Australia ( Delany *et al.*, 2014), and France ( Jabot *et al.*, 2020). What can be observed also over the past two decades is the enactment of innovative and creative ways of implementing HIA, in local and/ or national policy development, adopted across in many countries across the globe ( O'Mullane, 2013).

Current public health policies on the island of Ireland produced by the respective Departments (Ministries) of Health, namely *Healthy Ireland* (2013–2025) ( DoH, 2013) and *Making Life Better* (2013–2023) ( DHSSPS, 2013) endorse HIA as a way to facilitate this intersectoral, Health in All Policies, whole-of-government way of working for population health. In line with this endorsement, the all-island Institute of Public Health Ireland, published new HIA guidance in 2021 ( Pyper *et al.*, 2021), as part of a reinvigorated policy support for HIA across the island. Also, efforts in recent years in Ireland have sought to counter siloed ways of working for health and wellbeing. The *Healthy Ireland Strategic Action Plan 2021–2025* ( DOH, 2021) was launched in 2021, including the Sláintecare Healthy Communities Programme which puts in place a legal structure for local authorities to work in an intersectoral way with community agencies and health services for health and wellbeing improvement. This action plan aims to address health inequality in Ireland through an intersectoral Health in All Policies approach. *Healthy Ireland* recognises the relevance and importance of HIA in operationalising this intersectoral response. However, the *Healthy Ireland* action plan does not address the challenges of HIA implementation ( Gillespie & McIldoon, 2009; Kearns & Pursell, 2011) including the issue of how the approach could tangibly facilitate intersectoral action in tackling health inequalities. HIA-IM seeks to address these challenges through the co-creation of the HIA implementation model.

‘Development of a Health Impact Assessment Implementation Model: Enhancing Intersectoral Approaches in Tackling Health Inequalities’ (acronym: HIA-IM) is funded by the Health Research Board (HRB) under the Emerging Investigator Award (EIA), 2023 to 2026. Dr Monica O’Mullane holds this award as Principal Investigator, with team members involved in the project from across University College Cork (UCC), the Institute of Public Health Ireland, Cork Environmental Forum (CEF), the Environmental Protection Agency (EPA), Cork City Council, the Health Services Executive (HSE), Public Health Wales, Ben Cave Associates Insight, University of Galway, University of Bradford, University of New South Wales, Trinity College Dublin and University of Strathclyde. The HIA-IM Public Involvement Consultation Group includes members from Cork Environmental Forum, Cork Healthy Cities, Global Action Plan, Coalition 2030, Coast Watch Ireland, Global Health Ireland, Independent Living Group and Social Justice Ireland. The project is co-hosted in University College Cork between the School of Public Health and the Institute for Social Science in the 21 ^st^ Century (ISS21).

## Study aims and objectives

The aim of the project is to critically explore the process of developing a Health Impact Assessment implementation model that will enhance researcher, policy actor, practitioner, community, and voluntary sector capacity to collaboratively develop and implement intersectoral and equitable policy responses to major population health issues.

The project involves doing two HIAs as part of the research study, one at a local policy level, addressing the Cork City Development Plan (2022–2028) (HIA 1), and the second HIA at a national policy level, addressing the Irish Government’s Climate Action Plan (2024) (HIA 2). Simultaneously, the process of doing the HIAs will form the basis for the research study using an action research approach integrating implementation science theoretical frameworks. Research participants included in the study are the members of each of the two HIA Steering Groups (Group A), as well as individuals within key organisations involved in the HIAs with capacity to use and embed the practice HIA within existing structures (Group B). For the HIA Steering Groups, members are recruited from community organisations, health services, statutory environmental organisations, local authorities and the research/ academic community. The goal is that by drawing learning from participants’ lived experience and reflections across Groups A and B, we can connect evidence, policy, and practice in a co-created manner to directly inform the iterative building of the contextualised HIA implementation model. This model will be developed in a way to practically inform the conduct of HIA in practice through the roll out of the HIA implementation programme, led by the Institute of Public Ireland, as well as enhancing capacities and confidence of individuals in doing HIAs going forward. A key component of the HIA implementation model will be the creation of a Community Engagement Toolkit to facilitate meaningful community engagement, specific to the conduct of HIAs. Although the focus of the project is on HIA as one approach for implementing intersectoral action for improved population health, the exploratory nature of the work will reveal insight and nuances in the perceptions of the approach, in using the IPH guidance, and problematising HIA as a suitable approach for creating Health in All Policies within an Irish context.

Using revised Irish HIA guidance ( Pyper *et al.*, 2021), this mixed methods research study will employ action research approach integrated with implementation science theory to iteratively develop a contextualised HIA Implementation model, that will lead to implementing intersectoral and equitable policy responses in the future. HIA-IM will address five research objectives across four work packages which align with national public health policy priorities for population health in Ireland ( DHSSPS, 2013; DoH, 2013), with Northern Ireland ( DHSSPS, 2013), and internationally ( WHO, 2013). Through these objectives, HIA-IM will address the HIA implementation gap, as outlined in research on HIA implementation ( Gillespie & McIldoon, 2009) and national policy ( DoH, 2013; DOH, 2021).

The five research objectives, outlined below, align with four work streams. The four work streams are described in the methods section:

Research objective 1: Conduct two HIAs on (i) the Cork City Development Plan 2022–2028; (ii) the Government Climate Action Plan 2024

Research objective 2: Apply an action research cycle during the conduct of the two HIAs;

Research objective 3: Identify factors that influence the process of HIA implementation with a hybrid implementation science framework drawing from the Consolidated Framework for Implementation Research (CFIR) and Normalisation Process Theory (NPT);

Research objective 4: Produce a Community Engagement Toolkit in order to optimise community participation in HIA in Ireland

Research objective 5: Develop a contextualised contemporary Irish HIA implementation model iteratively by integrating and triangulating data from across the project.

### Study design

This research study will use multiple implementation science frameworks ( Damschroder *et al.*, 2022; Finch *et al.*, 2018) within an action research approach ( Bradbury, 2022) to co-produce in an iterative way a contextualised Health Impact Assessment implementation model with key partners. HIA implementation is optimised when relevant stakeholders contribute evidence and insight ( den Broeder *et al.*, 2017). HIA implementation with meaningful community engagement is integral to its sustainability as a public health response for tackling health inequalities, as has been evidenced in previous research ( Green *et al.*, 2021a). Using a community-centered and co-design approach, the first Community Engagement Toolkit for HIA in Ireland will be co-created with key partners. This Toolkit will be a practical resource to be used in carrying out participatory HIAs in Ireland, complementing the use of any HIA guidance. In using the HIA approach, the research study is founded on improving health equity for population groups who will be affected by the implementation of the policy under study, which in the case of HIA-IM, includes local urban policy and national climate action policy. The equity lens allows us to consider the health impacts of policies under study. The rationale, approach and timing of this research study is designed to inform the roll-out of the HIA implementation programme led by the Institute of Public Health, synergising with key national population health priorities and policy implementation going forward. Quantitative and qualitative methods will be employed underpinned by co-design and co-production approaches. A scoping review of factors influencing HIA implementation will be carried out in order to create research evidence that informs contextualised responses to create intersectoral policy for population health.

Action research has been shown to improve the contextualised nature of implementation procedures within organisational structures, compared to other research methods ( Bush *et al.*, 2017). However, little research has been conducted on the use of action research methods with implementation research, specifically in assessing implementation processes and outcomes of policies and policy-informing processes, such as HIA. Action research is applied in order to improve specific practices through critical reflection ( *ibid*). It is founded on participation and collaboration of individuals who work through an action research cycle which includes planning, acting, outcome, and reflection, to provide feedback (collected data) in order to introduce improvements to a specific practice. It presents a learning opportunity for data collection of participants’ reflections and learnings ( McNiff, 2013). Action research is an approach that co-creates research with people, places praxis and reflection as primacy, and is underpinned by social constructivism ( Bradbury, 2022). Hence, within this project, the research team adopts a critical sociological lens to allow space for reflections of social and power relationships with any nuanced implementation dynamics arising throughout the research, specifically in the conduct of the HIAs ( Ahmed, 2012; Nettleton & Bunton, 1995). The research team, who will participate in the two HIA Working Groups, will consciously embody a reflexive research role within the study in order to capture key reflections with participant observations, aligned in the spirit of meaningful action research ( Archibong *et al.*, 2016). Planned time and space for research analysis during data collection will take the form of two research analysis retreats for each HIA. This data will be triangulated with other research data produced within the study to create a holistic picture of HIA in Ireland, informing the resulting implementation model.

## Methods

### Work stream 1: Action research-led design and implementation of two HIAs using revised national HIA guidance. HIA 1 on the Cork City Development Plan (2022–2028) and HIA 2 and on the Irish Government Climate Action Plan (2024)

Two HIAs are planned within this research project, one on the Cork City Development Plan (2022–2028), one on the national Climate Action Plan (2024). Although containing the words ‘plan’ in their titles, both are policies, one operating at the local level, one in the national policy arena. The following outlines detail of the two policies, including their purpose, remit, geographic scope and population groups.

**Cork City Development Plan (2022–2028)**

The Cork City Development Plan (2022–2028) has a remit in creating a strategic spatial land-use policy for the city of Cork. The remit of the Plan is to guide development in the city across nine strategic objectives, including Compact Liveable Growth, Delivering Homes and Communities, Transport and Mobility, Climate and Environment, Green and Blue infrastructure, Open Space and Diversity, Economy and Employment, Heritage, Arts and Culture, Environmental Infrastructure, and Placemaking and Managing Development ( CCC, 2022). The geographic scope of the Plan is within the boundary of Cork city. The population of Cork city residents, comprising of 210,000 people ( *ibid*). 

**Climate Action Plan (2024)**

The national Climate Action Plan (2024) is the third annual update to Ireland’s Climate Action Plan ( GoI, 2024). The remit of the annual Plans is to provide a roadmap for taking decisive action to halve Ireland’s emissions by 2030 and reach net zero by no later than 2050. This commitment is included in the Climate Action and Low Carbon Development (Amendment) Act 2021. The Climate Action Plan (2024) builds on the previous iteration, published in 2023. It updates measures and actions that are required to deliver the carbon budgets and sectoral emissions ceilings. The geographic scope of the Plan is within the boundary of the Republic of Ireland. The population of Ireland comprises of 5,149,139 people, based on the latest census figures ( CSO, 2023).

***Task 1.1. Design and conduct a HIA on the Cork City Development Plan, 2022–2028 (HIA 1) and subsequently on the Government Climate Action Plan 2024 (HIA 2), using recently revised national HIA guidance***

The HIA Steering Groups, which will be established before each HIA commences, will steer the direction and scope of each HIA and carry out the work. Minutes from the Steering Group meetings, which will record matters arising and decisions made at each stage of the seven HIA stages, will be used as research data.

**Figure 1.**
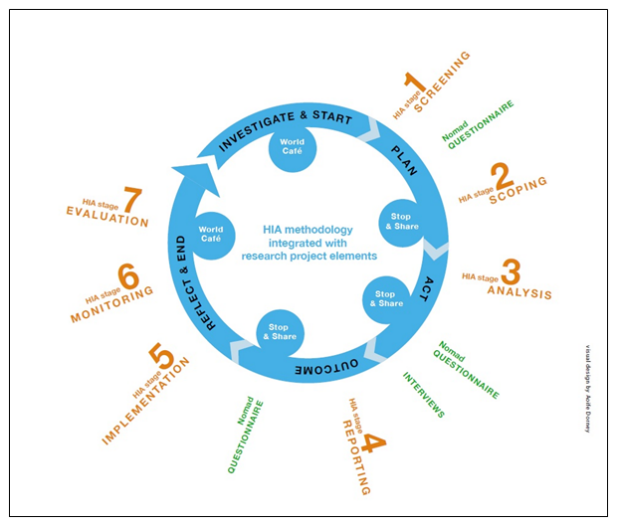
Proposed research framework, drawing on the action research cycle employed by Archibong *et al.* (2017).

In summary, the following processes will be implemented using HIA stagiest methodology as outlined in the IPH HIA guidance ( Pyper *et al.*, 2021) (orange stream in Figure 1):

**1.**  **Screening:** The screening tool will be applied to the national and local policies, to establish the range and distribution of potential impacts (p. 110).
**2.**  **Scoping:** The governance for the whole assessment process is established at this stage for each HIA. The scoping stage also decides on (based on screening tool output) the determinants of health and the populations to be assessed, as well as the methods by which they will be assessed (p. 40)
**3.**  **Analysis:** This is the most labour-intensive stage of the process. It involves the gathering, generating (when not available) and synthesis of quantitative and qualitative evidence of potential health impacts, the assessment of the distribution of those impacts (including potential impacts on health inequalities) and the work of drawing appropriate and relevant conclusions. The analysis involves an assessment according to guide questions (p. 47) as to the ‘likely’ and significant’ potential impacts, based on evidence. The analysis will draw on relevant sociodemographic data from the Central Statistics Office, including indices of deprivation mapped at small area (District Electoral Division) level - Pobal Maps and on population health data from the Healthy Ireland Outcomes Framework.
**4.**  **Reporting:** The HIA will report * a change to the policy and/or * a conclusion on particular effects, for example, that an effect is likely and significant.
**5.**  **Implementation:** At this stage, direct pathways will be identified by the steering groups for the HIA findings to be used in the two respective policies- City Development Plan and Government Annual Climate Action Plan
**6.**  **Monitoring:** Monitoring will track the actual effects and can be conducted during different phases of the policy. Part 6 of the Technical Guidance ( Pyper *et al.*, 2021) provides a tool for developing monitoring measures.
**7.**  **Evaluation:** A process and impact evaluation will be conducted at this stage of the HIA

***Task 1.2. Apply an action research cycle concurrent to carrying out HIA 1 and HIA 2 (Research objective 2)***

As illustrated in Figure 1 (blue stream) a five-stage action research cycle comprising Investigate, Plan, Act, Outcome and Reflect will be applied to the seven HIA stages ( Figure 1-orange stream) of HIA 1 and HIA 2. Such continual application of an action research cycle while Task 1.1 is delivered will ensure data from HIA processes are captured. This is a novel approach to researching HIA in practice, and will capture data, which is specific to the Irish process of HIA, thus informing the HIA implementation model.

Data on the implementation of both HIAs will be collected at each of the five action research cycle stages:

**1.**  ***Investigate***: Prior HIA stage 1 (Screening), participants’ reflections on the planned HIA process will be captured using a World Café forum. World Café is a participatory action research method widely used in organisational change processes, when engaging community and health care stakeholders. It is a conversational process, also termed a self-facilitating focus group, that helps groups to engage in constructive dialogue around critical questions, to build personal relationships, and to foster mutual learning ( Löhr *et al.*, 2020). It involves three rounds of conversation groups, recording results in the form of text, sketches, or symbols on paper. Through providing a forum of open informal dialogue and mutual learning, the World Café method tends to motivate participants. In addition to the World Café, data will be collected in the form of the research team’s observation notes.
**2.**  ***Plan***. HIA steering group participants’ perceptions and reflections on conducting the Screening and Scoping of HIA 1 and HIA 2 will be captured using a Stop & Share method ( Archibong *et al.*, 2016), an individual-level rapid interview method (20–30 minutes) A set of pre-defined prompts eliciting reflection on conducting the stages will offer an opportunity for participants to reflect individually.
**3.**  ***Act:*** HIA steering group participants’ perceptions and reflections on the Analysis stage of HIA 1 and HIA 2 will be captured by the research team in the form of observation notes and using the Stop & Share method.
**4.**  ***Outcome*** aligns with the stages 4 (Implementation) and 5 (Reporting) of the HIA process. Participant reflections will be collected using the Stop & Share method between stages 4 and 5. Therefore, this will capture participants’ experiences while writing the HIA report.
**5.**  ***Reflect***. A World Café will be conducted in order to capture participants’ collaborative reflections. It will be conducted in the same way as described for the first World Café. This further World Café will capture participants reflections in between the Monitoring and Evaluation stages of HIA 1 and HIA 2 (stages 6 and 7 of the HIA process).

A co-production workshop for data analysis is planned with key stakeholders including the project’s public involvement consultation group and the Institute of Public Health Ireland will take place. In line with action research ethos, this workshop will ensure key stakeholders will engage in the co-design of data analysis, ensuring that the people envisaged to work with HIA and use the resources of HIA-IM after project completion, will be involved in the research process. Incorporating the outcomes from this co-production of analysis, data will be further refined using the reflexive thematic analytical framework ( Braun & Clarke, 2022). Reliability checks will be used in the application of this project’s qualitative research methods ( Heikkinen *et al.*, 2012), including the inter-coder reliability measures to ensure the highest possible levels of consistency, validity and transparency in the data analyses.

### Work stream 2: Identification of factors that influence the process of HIA implementation, drawing from the Normalisation Process Theory (NPT) and the Consolidated Framework for Implementation Research (CFIR) (research objective 3) ( Figure 1, green stream)

HIA-IM will use multiple implementation science frameworks, drawing from the Normalisation Process Theory (NPT) ( Finch *et al.*, 2018) and the Consolidated Framework for Implementation Research (CFIR) ( Damschroder *et al.*, 2022). Relevant theoretical constructs from both frameworks will be used in order to analyse the **process** of doing or carrying out a HIA (NPT) and the **determining factors** affecting HIA implementation (CFIR). NPT comprises of four constructs; Coherence- what is the work?, Cognitive Participation- who does the work?, Collective Action- how does the work get done?, Reflexive Monitoring- how is the work understood? NPT in particular seeks to examine the implementation processes of complex interventions such as HIA, understood as deliberately institutionally sanctioned interventions that are formally defined; planned; and intended to lead to a changed outcome ( Finch *et al.*, 2018). NPT works to assess the implementation processes of those directly engaged in the intervention (HIA) as well as individuals not directly engaged but knowledgeable of the process ( *ibid*). CFIR comprises of five domains; intervention/ innovation characteristics, inner and outer setting, the individuals involved, process of implementation. In our research examining the implementation or doing of HIA in practice, the inner and outer settings domains of CFIR will be particularly illustrative in generating data on the wider institutional and policy contexts within which participants are working, enabling us to explore the relevant institutional and policy drivers and levers. CFIR was not developed to only focus on the institutional and policy context within which an intervention or approach such as HIA operates, however, the Outer Setting domain does include policy relevant attributes such as Policies and Laws, Local Conditions, and Local Attitudes that are relevant to the practice of HIA.

HIA 1 and HIA 2 steering group members from Task 1.1 will form Group A for work stream 2. Each HIA Steering Group structure will include 12 participants. Group B will comprise of individuals within key organisations involved in the HIAs with capacity to use and embed the practice HIA within existing structures (Group B). A snowballing technique will be used to identify and recruit Group B participants, estimated between 5 and 10 individuals per HIA (10 to 20 individuals in total).

An explanatory mixed-methods approach in data collection and analysis will be employed to capture implementation processes. Quantitative data will inform qualitative data collection and analysis, integrated sequentially with qualitative data ( Figure 1- green stream). The standardised validated NoMAD ( Finch *et al.,* 2018) questionnaire, with additional questions from CFIR domains, will be administered to Groups A and B. The NoMAD questionnaire assesses implementation processes from the perspective of professionals involved in the work of implementing complex interventions such as HIA.

Once the HIA stage of Scoping is completed, both groups A and B will be asked to complete the questionnaire online. They will be asked again after the HIA analysis stage, and following completion of the HIA. In total the groups will complete the questionnaire three times during the process of the HIA. Normalisation Process Theory (NPT), from which the NoMAD questionnaire is derived, addresses and highlights factors in the HIA process needed for successful implementation and integration of HIA into routine work (normalisation). Therefore, we administer the NoMAD questionnaire more than once during the HIA process, at the work-intensive points (Scoping, Analysis and Reporting), in order to capture the evolution, if any, of participant’s perceptions of factors needed to enhance HIA implementation.

Once the HIA process is completed, respondents from groups A and B will be invited to participate in semi-structured interviews. A purposive sample will be chosen, using selection criteria (organizational affiliation, decision-making authority, gender and intersectionalities balance), from those who volunteer for interviews. The interview schedules will incorporate the NPT and CFIR constructs as well as findings from the rounds of NoMAD questionnaire administration. This will allow the research team to elicit perceptions and experience on factors affecting the implementation of HIA from the perspective of individuals in Groups A and B.

### Work stream 3- Co-produce a Community Engagement Toolkit in order to optimise community participation in HIA in Ireland (research objective 4)

The aim of this work stream is to capture learning from the HIA Analysis stage (stage 3 of HIA process ( Figure 1) using a set of community engagement indicators, building on Frewer *et al.* (2000) criteria for effective public participation. The HIA Analysis stage includes community consultation, to inform the HIA with community knowledge. The goal is to counter potential community disenfranchisement from transformative policy change from going “over their heads,” as has been highlighted by Haigh *et al.* (2020: 1).

One of the four values central to HIA is democracy ( WHO, 1999). HIA enables a generation and synthesis of quantitative and qualitative evidence, including community knowledge. The *Healthy Ireland Framework 2013–2025* with its *Strategic Action Plan 2021–2025* both highlight the importance of Empowering People and Communities ( DOH, 2021; DOH, 2023) through intersectoral responses including HIA. However, HIA processes often lack meaningful community engagement ( den Broeder *et al*., 2017). As Haigh *et al.* (2020) conclude from their study of community engagement in HIA, this is identified as a missed opportunity to achieve benefits of community engagement during the assessment of planning processes that occur as part of HIAs. The Institute of Public health HIA guidance does not detail how community engagement could occur. HIA practitioners, scholars and advocates such as Green *et al.* (2021c) have demonstrated the importance of the community voice in developing sustainable HIA implementation.

A conceptual framework for community engagement will be built from the relevant theoretical and grey literature review, drawing on criteria for effective public participation for HIA ( Frewer *et al.*, 2000) and the published literature on the Health Equity Impact Assessment toolkit ( Povall *et al.*, 2013).

The review will draw on literature underpinning the rationale for community participation in HIA, identifying tools and practices that have been used to effectively facilitate communities in engaging in the HIA process, as well as carrying out their own HIA's. In addition, toolkits that have been developed to enhance community engagement will be included for review. The findings from the literature review will inform the conceptual framework for data collection for the HIAs. This framework will then be applied when involving communities in both HIAs, forming the conceptual content for data collection using the World Café method during the Analysis stage of the HIAs. Based on the findings from the application of the framework, which involves community participation during the Analysis stages of both HIAs, the Community Engagement Toolkit will be created. The Toolkit will be aimed at people who are doing HIAs and want guidance on community engagement, as well as guidance for community groups on engaging in HIAs. Data for work stream 3 will be drawn from work streams 1 and 2 of community representatives on the HIA Steering Groups, as well as part of the Analysis stage of the HIA.

### Work stream 4: Iterative development of a contextualised, contemporary, Irish HIA implementation model by integrating and triangulating qualitative and quantitative data from all work streams (research objective 5)

Identified limitations in the literature will be addressed in HIA-IM including a scoping review of factors affecting HIA implementation, with a view to informing the development of the HIA implementation model. This scoping review will inform the integration of data from all work streams as well as the main output from the research study, the HIA implementation model. Contributions from the Public Involvement in the research study is crucial to the development of the HIA implementation model. This work stream 4 involves four Public Involvement events with the HIA-IM Public Involvement Consultation Group, in the first three years of the project, in order to gather contributions to inform the development of the model. In the fourth and final year of the project, a Public Forum event will be held in order to include further contributions from the public to the model, beyond the Consultation Group. This will ensure the model is both informed by scientific evidence together with contributions from the Public Involvement process.

Upon completion of both HIA 1 and HIA 2, data will be synthesised and analysed in order to iteratively build the contextualised Irish HIA implementation model. The research study will do this through a process that documents the implementation process in the conduct of two HIAs. Work stream 4 will consolidate the findings on impact and effectiveness of HIA 1 and HIA 2 (Task 1.1), themes emerging from Task 1.2, findings from work stream 2, findings from the creation of the Community Engagement Toolkit (work stream 3) and four PPI events (work stream 4) in order to produce the contextualised HIA implementation model.

Integration of data occurs at three stages in HIA-IM. The model will be produced iteratively as findings from HIA 1 will be integrated with findings from HIA 2 creating the report on the application of the action research cycle to HIA implementation. Integration will take place at the interpretation stage of the study, to triangulate findings. Specifically, triangulation will be carried out during the iterative and continual synthesis and integration stages, where a convergence coding matrix will be developed to display findings emerging from all work streams.

A central feature of the research study will be to ensure that research findings and outcomes are interpreted by the research team, staff of the Institute of Public Health Ireland (IPH), and relevant collaborators during research team, Steering Group and Advisory Panel meetings, and meetings with project collaborators. Contributions at PPI events will facilitate contributions to identify key priorities and recommendations, as well as co-production in data analysis, informing the model. The main outcome from work stream 4 will be the continual development of the HIA implementation model, which will be built iteratively and consistently throughout the project. This model will be populated by data findings from all work streams.

The HIA implementation model will include an explanation of the implementation of HIA at local policy level and national policy level, in order to uncover, enhance understanding and offer a guiding tool and process for doing HIAs in Ireland, at the policy, strategic level.

## Results dissemination

As the action research approach is central to the study, results dissemination will be done in an iterative way during the conduct of both HIAs, with ongoing dissemination and learning to be carried out with both of the two HIA Steering Groups. There will be a variety of research dissemination outputs resulting from this research project including peer-reviewed journal articles, policy briefs, research papers, published dataset with the Irish Qualitative Data Archive (IQDA), and conference proceedings as a result of this research project. The translation of evidence to policy impact pathway inherent in the partnership between project team and leading health policy actors on the island of Ireland, namely the Institute of Public Health who are leading on the roll-out of the HIA programme and within the HSE, will influence the implementation of HIA on the island of Ireland. These publications including the Community Engagement Toolkit will be disseminated through the School of Public Health and ISS21 websites, the HIA Public Involvement Consultation Group members, project partners, team members and academic networks. The two HIA reports, and the Community Engagement Toolkit, will be stored on the University College Cork open access repository (CORA). Results will be reported according to the COREQ guidelines as appropriate ( Booth *et al.*, 2014). The results of the project may inform action within the public health sector to consider a One Health approach in tackling the polycrisis for all living organisms affected by climate change, environmental health, biodiversity and a circular economy as important factors affecting global health going forward.

## Study status

This research is currently underway.

## Discussion

This research study is carried out using the expertise and experience of the PI and project team, who have a track record of high-quality research in HIA, implementation science, action research methodologies, environmental sociology, public activism, and Health in All Policies. The way in which the PI and project team have worked together has been collaborative from the start, designing the work programme in an iterative and consensual way before the project commenced and continuously, with key partners. Co-creation and co-production of the HIA on the Cork City Development Plan (2022–2028), of the HIA on the national Climate Action Plan (2024), Community Engagement Toolkit and HIA implementation model is at heart of the study’s work programme. Partnership working and co-creation across the research study will enhance understanding of HIA implementation processes and uncover the nuanced experience and power dynamics arising in the conduct of HIA. It will provide a guiding tool with the HIA implementation model to strengthen intersectoral approaches in tackling health inequalities and optimise health equity, as well as contribute to academic literature in the field.

## Ethical approval and consent

Ethical approval was sought from the University College Cork, Social Research Ethics Committee (SREC) in May 2023, and secured in June 2023 (Ethics approval number: 2023-091). An ethics amendment was approved by SREC in December 2023, which included detail on the data collection instruments and a description of the Public Involvement process engaged in the project. This study protocol will be sent to SREC in UCC for their information and records. Primary data will be derived from interviews, questionnaires, World cafés, HIA group meeting minutes and researcher reflections. Participant information sheets, consent forms and Conflict of Interest statements were approved during the UCC ethics approval process.

## Data Availability

No data are associated with this article.
