# Supplementary material for: Development of a Health Impact Assessment Implementation Model: Enhancing Intersectoral Approaches in Tackling Health Inequalities- A Mixed Methods Study Protocol
Source: HRB Open Res. 2025 Feb 6;7:14. Originally published 2024 Mar 20. [Version 3] doi: 10.12688/hrbopenres.13873.3 (PMC11862365; doi:10.12688/hrbopenres.13873.3)
Supplement: Peer review history

# Reviewer report: Reviewer response for version 3

**DOI:** 10.21956/hrbopenres.15466.r45418

**Published:** 2025-02-25

**Authors:** Morufu Olalekan Raimi

**Affiliations:** 1 Federal University Otuoke, Otuoke, Bayelsa, Nigeria

**HRB Open Research**

 The theme “Development of a Health Impact Assessment Implementation Model” is an essential contribution to public health and policy implementation. The study aligns with Health in All Policies (HiAP) and intersectoral governance models. Also, the integration of action research and implementation science frameworks by global HIA best practices and frameworks. The research has direct applications in policy implementation and public health interventions. Although, the model could be adapted for other countries looking to strengthen HIA frameworks. Using AI-driven data analysis could enhance HIA implementation and scalability and the study can inform urban planning, climate policy, and social justice initiatives. However, the manuscript requires several improvements for a clearer and stronger impact.

- The title is informative, appropriate, clear and concise, effectively summarizing the research focus. However, it will be better featured as “A Health Impact Assessment Implementation Model for Policy Integration.”
- The abstract is well-structured. however, the abstract should emphasize how this model advances existing HIA practices.

- Rationale: The abstract outlines the importance of HIA but does not adequately highlight the limitations of existing models.
- Objective: Clearly stated, but it should mention how the study builds on previous HIA research.
- Methods: The methodology is well-articulated, but more details on the research frameworks used (e.g., CFIR, NPT) should be included. The proposed model is not tested in multiple policy environments to verify its robustness.
- Results: The abstract should provide a more structured summary of the key innovations of the model.
- Conclusions: Should emphasize the applicability of the model to different policy settings.
- Recommendations: The abstract should outline how policymakers can apply this model in practice.

- Keywords are appropriate
- The introduction provides good background and contextualizes HIA within health policy but needs a clearer statement of research gaps.
- The manuscript should better articulate why previous HIA models have been insufficient.
- Author should avoid First-Person Pronouns: The author should replace “we” with “this study” or “the present study.”
- Methodology - More details on participant recruitment and selection criteria should be included. The methodology should elaborate on data triangulation techniques to enhance validity.
- Ethical considerations should be expanded, especially regarding community participation.
- Results - Clearly structured and well-detailed with model development process.
- Needs stronger comparisons with international HIA models.
- Author should further discuss barriers to implementation and real-world applicability.
- Figures need to better illustrate the implementation model.
- Tables should be referenced more frequently in the text.
- Discussion - The manuscript does not sufficiently discuss political and institutional barriers to HIA adoption. The study would also benefit from comparisons with HIA models in different policy contexts (e.g., EU vs. North America).
- There are few grammatical errors and spelling mistakes in this article. The author should limit the use of personal pronounces and the use of “we” and “our” in the article. Use third person pronoun and past tense in the write up of the article.  Reread this article for typographical, grammar and spelling errors.
- Heavy Use of Technical Language – The manuscript could be clearer for a multidisciplinary audience.
- Conclusion – Well-structured but should include policy implications.

- Author needs a stronger statement on policy recommendations and long-term monitoring.

- References – Comprehensive but needs more international case studies. Need more recent citations (2020–2024) and ensure consistent formatting (APA/Harvard style).

Specific Reviewer Comments and Suggestions:

1. Please give a frank account of the strengths and weaknesses of the article (in 100-200 words)

Is the study design appropriate for the research question?

Yes

Is the rationale for, and objectives of, the study clearly described?

Yes

Are sufficient details of the methods provided to allow replication by others?

Partly

Are the datasets clearly presented in a useable and accessible format?

Not applicable

Reviewer Expertise:

Health Impact Assessment; Environmental Impact Assessment; Environmental Epidemiology; Climate Change and Health; Medical Geography; Pollution Control Management; Waste Management and Health etc.

I confirm that I have read this submission and believe that I have an appropriate level of expertise to confirm that it is of an acceptable scientific standard, however I have significant reservations, as outlined above.

# Reviewer report: Reviewer response for version 3

**DOI:** 10.21956/hrbopenres.15466.r45402

**Published:** 2025-02-25

**Authors:** Courtney L McNamara

**Affiliations:** 1 Newcastle University, Newcastle upon Tyne, England, UK

The authors' revisions have addressed my concerns, and I am happy to update the status of the article to approved without reservations.

Is the study design appropriate for the research question?

Yes

Is the rationale for, and objectives of, the study clearly described?

Partly

Are sufficient details of the methods provided to allow replication by others?

Partly

Are the datasets clearly presented in a useable and accessible format?

Not applicable

Reviewer Expertise:

NA

I confirm that I have read this submission and believe that I have an appropriate level of expertise to confirm that it is of an acceptable scientific standard.

# Reviewer report: Reviewer response for version 2

**DOI:** 10.21956/hrbopenres.15391.r44395

**Published:** 2025-01-29

**Authors:** Marleen Bekker

**Affiliations:** 1 Wageningen University & Research, Wageningen, Gelderland, The Netherlands

The detailed response and the summary it looks like the authors have sufficiently processed the comments and suggestions in the new version.

Is the study design appropriate for the research question?

Yes

Is the rationale for, and objectives of, the study clearly described?

Partly

Are sufficient details of the methods provided to allow replication by others?

Partly

Are the datasets clearly presented in a useable and accessible format?

Not applicable

Reviewer Expertise:

NA

I confirm that I have read this submission and believe that I have an appropriate level of expertise to confirm that it is of an acceptable scientific standard.

# Reviewer report: Reviewer response for version 1

**DOI:** 10.21956/hrbopenres.15209.r38902

**Published:** 2024-05-20

**Authors:** Courtney L McNamara

**Affiliations:** 1 Newcastle University, Newcastle upon Tyne, England, UK

The manuscript ‘Development of a Health Impact Assessment Implementation Model: Enhancing Intersectoral Approaches in Tackling Health Inequalities- A Mixed Methods Study Protocol’ discusses the development and implementation of a Health Impact Assessment (HIA) model aimed at enhancing intersectoral approaches to tackle health inequalities in Ireland. It employs a mixed methods research strategy incorporating action research and implementation science theories to develop a contextualized HIA Implementation Model. The study is structured around several work streams that address five objectives and includes the production of a Community Engagement Toolkit and dissemination strategies.

 Overall, the study addresses a topical/pressing issue, using a robust methodological approach. The manuscript, however, is structured and written in a manner that might be confusing for readers. The transitions between sections are not always clear, and it can be challenging to discern the main objectives and rationale of the study’s methods.

 The study’s aim for example is discussed in several places using different terminology. The authors might consider some restructuring so that that the aim (mentioned just once) flows directly from an introduction that introduces the research gap, followed by methods and results/conclusions. It would be helpful when outlining the research gap to also describe the rationale for action research and implementation science strategies broadly and for the specific frameworks that are being used.

 Please check the numbering/characterization of the objectives on page 4 as the numbering system at the moment is a bit confusing and seems to list only 4 objectives.

 Greater detail should be provided on the steering and working groups and who will be included in them.    

 Although the creation of a Community Engagement Toolkit is mentioned, there doesn’t seem to be a public component to the dissemination strategy.

 Minor recommendation

 P 3  The following sentence: Employment status and education is associated with people reporting ‘good or very good health’ in the Healthy Ireland survey (DOH, 2023; Duffy et al., 2022).”

 Should be revised to mention that *those who are employed and higher educated* are more likely to report 'good or very good' health

Is the study design appropriate for the research question?

Yes

Is the rationale for, and objectives of, the study clearly described?

Partly

Are sufficient details of the methods provided to allow replication by others?

Partly

Are the datasets clearly presented in a useable and accessible format?

Not applicable

Reviewer Expertise:

My background is in public health and health equity and I have experience with HIAs

I confirm that I have read this submission and believe that I have an appropriate level of expertise to confirm that it is of an acceptable scientific standard, however I have significant reservations, as outlined above.

# Response

**Published:** 2024-12-13

**Authors:** Monica OMullane

**Affiliations:** Public Health, University College Cork, Cork, County Cork, Ireland

Dear Courtney, Many thanks for this review. 

 The Aim mentioned now only in the ‘study aims and objectives section,’ following the introduction that introduces the research gap.

 Rationale for using implementation science and action research in the introduction, as well as rationale for why using the two IS frameworks.

 Under the ‘Study aims and objectives section,’ the five research objectives are now listed.

 Under the section ‘study aims and objectives,’ I’ve included details on the broad sectoral representation of who is on the steering groups.

 This information is included now in the ‘results dissemination’ section- that the HIA reports, and the Community Engagement Toolkit will be stored on an open access repository and will be disseminate across networks including the HIA-IM Public Involvement Consultation Group.  

 Amended the sentence in the introduction section of the Protocol to read:

 Those who are employed and higher educated are more likely to report 'good or very good' health ( DOH, 2023; Duffy *et al.*, 2022).

# Reviewer report: Reviewer response for version 1

**DOI:** 10.21956/hrbopenres.15209.r38898

**Published:** 2024-04-13

**Authors:** Marleen Bekker

**Affiliations:** 1 Wageningen University & Research, Wageningen, Gelderland, The Netherlands

The paper 'Development of a Health Impact Assessment Implementation Model: Enhancing Intersectoral Approaches in Tackling Health Inequalities- A Mixed Methods Study Protocol' offers a commendable approach to experiment with and reflect on implementation conditions for HIA and its conclusions and policy recommendations. Identifying an implementation gap of HIA into intersectoral policies and actions addressing health inequalities, the study sets out to experiment with a local and a national level HIA, using these as action research settings for exploring theory-driven as well as experience-based implementation conditions and co-developing with study participants an implementation model and a Community Engagement Toolkit. The authors argue that this design will also allow for critical assessment of whether HIA overall is fit for purpose.

 The action research approach in my view offers important conditions to do justice to observed implementation barriers such as reflexive dialogues with HIA actors, affected groups and end users moving from evidence to meaning through joint sense-making and shared value creation, developing a common language and ambition horizon, as well as developing a shared understanding and sense of acceptability, feasibility and applicability of policy recommendations within the institutional frames and boundaries of voluntary coordination in that particular policy field. Actions that move beyond these boundaries require alternative strategies such as activism, advocacy or litigation. The reflection-in-action design could however take a broader approach, moving the development of a toolkit and a model towards developing conditions for a sustainable organizational infrastructure collecting policy feed forward and feedback for policy responsiveness and change, of which HIA can be an important partial component. This could institutionalize ownership in the non-health policy domain. It is of crucial importance to understand that implementation is not in the hands of HIA proponents, and ownership needs to be organized beyond the HIA.

 On a more critical note, the study design reads as a rather technical approach to procedures of HIA and action research. Yet the study purposes of policy implementation and change are fundamentally political - whether at the micro, meso or macrolevel of government and its relations to society. Therefore the study design could benefit from adding an explicit framework for the analysis of power within and between governmental and societal actors and stakeholders. Understanding the political dynamics allows HIA proponents to leverage the access, voice and veto options in the political system in strategies additional to HIA to the benefit of the public's health. Why would this be important? Because HIA implementation cannot be enforced. We need to deeply understand the mechanisms of policy change apart from HIA in order to devise appropriate strategies.

 Under policy and institutional conditions by which intersectoral policies and HIAs are 'endorsed' - and therefore in the absence of regulatory obligations, sanctions and enforcement, the coordination of such HIAs and their policy recommendations depends largely on voluntary mechanisms.Voluntary policy coordination and alignment across sectors, although often described as 'weak' or 'soft' mechanisms, rely on an ever growing repertoire of strategies, instruments, arrangements and networks that can produce strong and hard policy change to better promote and protect the health of affected groups or populations. However, such policy change usually does not result from a singular HIA but rather from intentional or unintentional combinations of strategies that significantly broaden the relevance to a larger and more powerful group, external events that induce contextual changes in public or political support, and/or shifts in power within the bureaucracy or in the political system. Any HIA initiative therefore could be more embedded in an approach to understand and act upon the complex adaptive system around the policy at hand. Secondly, HIA could become embedded in a sustainable learning infrastructure that connects operational, instrumental policy learning within a single HIA to organizational learning and adaptation of policymaking rules and procedures and to system level learning and change. The system level incorporates an infrastructure built around governmental monitoring and evaluation that makes policies more responsive to issues of legitimacy. Deeply understanding the politics of, for instance, permit procedures, practices, lobbying and (lack of) enforcement puts HIA in a different perspective. It might be helpful to use the action research design to develop a decision tool on what strategies would fit a variety of health impacts and purposes. 

 It might be interesting to further strengthen this study protocol in the literature with alternative  considerations of the Health FOR All Policies model and a co-benefits approach towards a persuasive argumentation for policy change; and whole of society approaches for the convergence of health and economy - an anticipatory rather than reactive approach to state-society partnerships. 

 Some suggestions concern specific points in the use of theory and methods, these are listed here.

 On the use of theory: the Normalisation Process Theory (NPT) and the Consolidated Framework for Implementation Research (CFIR): why and how did the authors select these two and how do these complement each other? The CFIR was not developed with a focus on public policies but, rather, to address organizational or professional policies that do not have to comply with or are not set according to democratic requirements. 

 Methods

 - Although participation of key implementation actors and end users is mentioned under the Analysis stage of HIA, it is of crucial importance that implementation actors are already part of the study from the very first moments of designing the HIAs in the Steering and Working Groups.

 - The protocol states that a conceptual framework for meaningful community engagement needs to be developed. The World Café offers an organizational procedure that might be enriched with for instance the technique of responsive evaluation (first developed by Robert Stake). Here the dialogue is owned and designed by participants. It focuses on sharing and understanding their issues, claims and concerns, articulating the controversies and avoiding premature consensus. This has been evaluated and proven highly effective in communities and stakeholders feeling heard, developing common understanding and offering increased engagement and alternative actions. 

 - I would suggest to replace the Stop and Share interview during the reporting and implementation stage with this World Café format since this seems a crucial stage to the study's purpose. Moreover, participants learn from each other enriching the model further.

 - I am unfamiliar with the NoMAD questionnaire and it is unclear to me why participants would have to fill out this questionnaire three times, since this study does not have an experimental or longitudinal setup.

 - the study protocol does not elaborate on the Cork City Development plan nor the national Climate Action plan. It is therefore unclear what specific conditions are relevant for the HIA as well as the action research design, and to what extent the implementation model and citizen engagement kit are generalizable to other policy proposals, geographic regions and population groups. 

 - The protocol does not address a theoretical framework for health inequalities. The design might benefit from using scientific publications on the Health Equity Impact Assessment toolkit.

 - The study organization with for instance the Public Involvement Group is convincing to ensure co-creation and co-production with relevant stakeholders and community representatives. The quality however will depend on who will participate and the range of diversity represented. This is not specified yet.

 Overall the protocol addresses an important and  increasingly urgent issue of aligning policies to protect public health. However, the protocol is executed in a rapidly changing context with increasing uncertainties and pressures on democratic and governance stability. Moreover,  climate change increasingly shows how human health in the complex ecosystem depends on environmental and planetary conditions. As a last suggestion, it might not suffice to focus the HIA implementation model and the community engagement toolkit on human health and human inequalities alone. The public health sector itself might consider going beyond human health to incorporate other living organisms affected by climate change, environmental health, biodiversity, and a circular economy as crucial risks and resources to human health. 

 Refer:

(PDF) The Politics of Healthy Policies: Redesigning health impact assessment to integrate health in public policy (researchgate.net)

Is the study design appropriate for the research question?

Yes

Is the rationale for, and objectives of, the study clearly described?

Partly

Are sufficient details of the methods provided to allow replication by others?

Partly

Are the datasets clearly presented in a useable and accessible format?

Not applicable

Reviewer Expertise:

Transformative health governance; Health policy and politics; Global One Health

I confirm that I have read this submission and believe that I have an appropriate level of expertise to confirm that it is of an acceptable scientific standard, however I have significant reservations, as outlined above.

## References

[1] : From policy coherence to 21st century convergence: a whole-of-society paradigm of human and economic development. *Ann N Y Acad Sci* .2014;1331: 10.1111/nyas.12511 201-215 10.1111/nyas.12511 25146105

[2] : Responsive evaluation in health promotion: its value for ambiguous contexts. *Health Promot Int* .2005;20(4) : 10.1093/heapro/dai013 391-7 10.1093/heapro/dai013 15972302

[3] : Implementation of the Health Equity Impact Assessment (HEIA) tool in a local public health setting: challenges, facilitators, and impacts. *Can J Public Health* .2020;111(2) : 10.17269/s41997-019-00269-2 212-219 10.17269/s41997-019-00269-2 31755052 PMC7109223

[4] : Political analysis in public health: middle-range concepts to make sense of the politics of health. *European Journal of Public Health* .2018;28(suppl_3) : 10.1093/eurpub/cky159 3-6 10.1093/eurpub/cky159

[5] : From Health in All Policies to Health for All Policies. *Lancet Public Health* .2022;7(8) : 10.1016/S2468-2667(22)00155-4 e718-e720 10.1016/S2468-2667(22)00155-4 35907422 PMC9330081

# Response

**Published:** 2024-04-18

**Authors:** Monica OMullane

**Affiliations:** Public Health, University College Cork, Cork, County Cork, Ireland

Many thanks for your review and insightful comments and report.

 The research is set within the context of the Health for all Policies realm, which we can include explicitly in the protocol. The setting theoretical domain of CFIR will capture the broader institutional and political context, and the extent to which is does that, will be reflected by us in the findings.

 The research seeks to examine implementation processes, with a particular focus on the process of implementation, which NPT can theoretically capture, and the factors determining or shaping implementation of a specific approach, such as HIA, which is the validity of using CFIR. Both are well established implementation science frameworks and are often used together (for example- this article [Schroeder, D., Luig, T., Finch, T.L. *et al.* Understanding implementation context and social processes through integrating Normalization Process Theory (NPT) and the Consolidated Framework for Implementation Research (CFIR). *Implement Sci Commun* **3**, 13 (2022). https://doi.org/10.1186/s43058-022-00264-8] looking to understand implementation context with CFIR and NPT). In the protocol, we can revise and clarify why we are using these chosen frameworks, and why together. Using these frameworks with HIA in mind as an innovative process is novel, and we seek to illustrate how and why the frameworks were useful, and also, if or when they were not applicable.

 In response to queries regarding Methods used:

- We are currently in the middle of data collection and therefore cannot change the Stop & Share for World Café; however, point duly noted for when planning HIA 2. What we are finding so far with doing the Stop & Shares at the various stages of the HIA is that there are revealing aspects of subjective experience of the process of the HIA that participants are reflecting in an open manner and this space seems to work well in their enabling their reflection during the ‘doing’ of the HIA.
- NoMAD is a standardised survey developed from the domains of NPT; the reason for administering it three times during the process of data collection is to elicit data on how participants are experiencing the process of HIA at various stages of the HIA. Although not a longitudinal study, we are interested to see if there are any changes in the groups experience and involvement in the HIA.
- We seek to detail the policies under study when we publish results. In these further papers and in the publication of the implementation model, we will detail to which extent is the protocol and subsequent model with toolkit generalizable to other policies, geographic regions and population groups.
- We will review the design of the Community Engagement Toolkit to include design with scientific publications on the Health Equity Impact Assessment toolkit.

# Response

**Published:** 2024-12-13

**Authors:** Monica OMullane

**Affiliations:** Public Health, University College Cork, Cork, County Cork, Ireland

More detail on responding to the review: 

 We will make it explicit in the protocol that when we refer to the implementation of HIA, we are referring to the doing of HIA in practice: This is included now in the Introduction, and in the Methods section.

 Under work stream 2 in the ‘methods section, I’ve made explicit that we are looking at HIA implementation with our research approach and using the outer and inner settings domains of CFIR to explore relevant policy or institutional drivers or levers.

 Included Health for All Policies reference in the ‘introduction,’ where HiAP is first mentioned.

 Included in the manuscript an explanation as to why we chose the two IS frameworks, why and how they complement each other. This is now in the Introduction section.

 Included in the Methods section, in the Work Stream 2 section: I would argue that whilst yes, CFIR was not developed solely to focus on public polices, one of its five domains (Damschroder et al. 2022) in the originla and updated CFIR includes the outer setting, which includes policy relevant attributes such as Policies & Laws, Local Conditions, Local Attitudes and so on.

 The information on Steering and Working Groups is included in the Methods section, under work stream one, task 1.1.

 The reference for the NoMAD survey is  included in the protocol (Finch et al. 2018), from my searching online, the Finch et al 92018) paper looks like the accepted published paper on the NoMAD survey/ questionnaire https://implementationoutcomerepository.org/implementation-outcomes/sustainability/normalisation-measure-development-questionnaire-nomad

 Information included in the Methods section under Work Stream 2 in relation to rationale for repeating the administration of the survey. 

 More detail is provided on the two policies, namely, the Cork City Development Plan (2022-2028) and the Climate Action Plan (2024), specifying their remit and scope, geographic scope, and population groups.

 Included now in the section:

 Included the Health Equity Impact Assessment to the Work stream 3- Section.

 Members of the HIA-IM public Involvement Consultation Group are included in the Introduction.  Included a point on this in ‘results dissemination.’

 I include reference to Bekker (2007) also.
